# Robust clustering in high dimensional data using statistical depths

**DOI:** 10.1186/1471-2105-8-S7-S8

**Published:** 2007-11-01

**Authors:** Yuanyuan Ding, Xin Dang, Hanxiang Peng, Dawn Wilkins

**Affiliations:** 1 Computer & Information Science Department, The University of Mississippi, University, MS, USA; 2 Department of Mathematics, The University of Mississippi, University, MS, USA

## Abstract

**Background:**

Mean-based clustering algorithms such as bisecting *k*-means generally lack robustness. Although componentwise median is a more robust alternative, it can be a poor center representative for high dimensional data. We need a new algorithm that is robust and works well in high dimensional data sets e.g. gene expression data.

**Results:**

Here we propose a new robust divisive clustering algorithm, the *bisecting k-spatialMedian*, based on the statistical spatial depth. A new subcluster selection rule, *Relative Average Depth*, is also introduced. We demonstrate that the proposed clustering algorithm outperforms the componentwise-median-based bisecting *k*-median algorithm for high dimension and low sample size (HDLSS) data via applications of the algorithms on two real HDLSS gene expression data sets. When further applied on noisy real data sets, the proposed algorithm compares favorably in terms of robustness with the componentwise-median-based bisecting *k*-median algorithm.

**Conclusion:**

Statistical data depths provide an alternative way to find the "center" of multivariate data sets and are useful and robust for clustering.

## Background

In gene expression studies, the number of samples in most data sets is limited, while the total number of genes assayed is easily ten or twenty thousand. Such high dimension and low sample size data arise not only commonly in genomics but also frequently emerge in various other areas of science. In radiology and biomedical imaging, for example, one is typically able to collect far fewer measurements about an image of interest than the number of pixels.

These HDLSS data present a substantial challenge to many methods of classical analysis, including cluster analysis. In high dimensional data, it is not uncommon for many attributes to be irrelevant. In fact, the extraneous data can make identifying clusters very difficult [1]. Robust clustering methods are needed that are resistant to small perturbations of the data and the inclusion of unrelated variables [2].

The bisecting *k*-means algorithm is a hybrid of hierarchical clustering and the *k*-means algorithm. It proceeds top-down, splitting a cluster into two in each step, after which it will select one cluster based on a selection rule (commonly the cluster with the largest variance) to further split. In each splitting step, it randomly picks a pair of data points that are symmetric about the "center" of the data and assigns all other data points to one cluster or the other based on distance to the two selected points, thus the algorithm is similar to the *k*-means algorithm. The center is usually the mean. This whole process continues until each point is a cluster or a predefined number of clusters is reached.

Similar to other commonly used methods that are based on mean, e.g. *k*-means, bisecting *k*-means is not robust because the mean is susceptible to outliers and noise [3]. As a common remedy, the bisecting *k*-median algorithm, which replaces the mean by the componentwise median, is less sensitive to outliers. However, the componentwise median may be a very poor center representative of data, because it disregards the interdependence information among the components and is calculated separately on each component (dimension). For example, the componentwise median of the points (*a*, 0, 0), (0, *b*, 0) and (0, 0, *c*) for arbitrary reals *a*, *b*, *c *is (0, 0, 0) which even does not lie on the plane passing through the three points.

A new center representative for multivariate data that is robust and takes into account the interdependence among the dimensions is clearly needed.

Of the various multivariate medians, however, those defined via statistical depth functions are advantageous because the theory of statistical depth has been quite nicely established, though it is still relatively young and still under development. Analogous to linear order in one dimension, statistical depth functions provide an ordering of all points from the center outward in a multivariate data set. Linear order induces an ordering and ranking for 1-dimensional observations. Median is the "deepest" point in the data set. In contrast, for dimension *d *≥ 2, there is no natural order. As compensation, it is convenient and natural to orient to a "center", the deepest point, that is, the multivariate median. This leads to center-outward ordering of points and to a description in terms of nested contours. Tukey [4] first introduced halfspace depth. Oja [5] defined Oja depth. Liu [6] proposed simplicial depth. Zuo and Serfling [7] considered projection depth. Other notions include Zonoid depth [8], generalized Tukey depth [9], and spatial depth [10] among others. See [7] for a systematic exhibition.

Of the various depth functions, the *spatial depth *is especially appealing because of its computational ease and mathematical tractability, see Vardi [11], Serfling [12], Chaudhuri [10] and Koltchinskii [13] among others. The spatial depth (SPD) of a point *x *w.r.t. a distribution *F *is defined as

SPD(*x*, *F*) = 1 - ||E
 MathType@MTEF@5@5@+=feaafiart1ev1aaatCvAUfKttLearuWrP9MDH5MBPbIqV92AaeXatLxBI9gBaebbnrfifHhDYfgasaacH8akY=wiFfYdH8Gipec8Eeeu0xXdbba9frFj0=OqFfea0dXdd9vqai=hGuQ8kuc9pgc9s8qqaq=dirpe0xb9q8qiLsFr0=vr0=vr0dc8meaabaqaciaacaGaaeqabaqabeGadaaakeaatuuDJXwAK1uy0HMmaeHbfv3ySLgzG0uy0HgiuD3BaGabaiab=ri8fbaa@388C@_*F*_*S*(*x *- *X*)||, *x *∈ ℝ^*d*^,

where *S*(*x*) = *x/*||*x*|| is the spatial sign function (*S*(0) = 0) with Euclidean norm ||·||. The sample spatial depth is

SPD(x,Fn)=1−‖1n∑i=1nS(x−Xi)‖,x∈ℝd,
 MathType@MTEF@5@5@+=feaafiart1ev1aaatCvAUfKttLearuWrP9MDH5MBPbIqV92AaeXatLxBI9gBaebbnrfifHhDYfgasaacH8akY=wiFfYdH8Gipec8Eeeu0xXdbba9frFj0=OqFfea0dXdd9vqai=hGuQ8kuc9pgc9s8qqaq=dirpe0xb9q8qiLsFr0=vr0=vr0dc8meaabaqaciaacaGaaeqabaqabeGadaaakeaafaqabeqacaaabaacbaGae83uamLae8huaaLae8hraqKae8hkaGccbiGae4hEaGNae4hlaWIae4Nray0aaSbaaSqaaiab+5gaUbqabaGccqGGPaqkcqGH9aqpcqaIXaqmcqGHsisldaqbdaqaamaalaaabaGaeGymaedabaGaemOBa4gaamaaqahabaGaem4uamLaeiikaGIaemiEaGNaeyOeI0IaemiwaG1aaSbaaSqaaiabdMgaPbqabaGccqGGPaqkaSqaaiabdMgaPjabg2da9iabigdaXaqaaiabd6gaUbqdcqGHris5aaGccaGLjWUaayPcSdGaeiilaWcabaGaemiEaGNaeyicI48efv3ySLgznfgDOjdaryqr1ngBPrginfgDObcv39gaiqaacqqFDeIudaahaaWcbeqaaiabdsgaKbaakiabcYcaSaaaaaa@5F4B@

where *F*_*n*_(*x*) is the empirical distribution function of the data *X*_1_,...,*X*_*n*_. Points deep inside the data cloud have high depth values, while the points on the outskirts have lower depth values.

**Figure 1:**
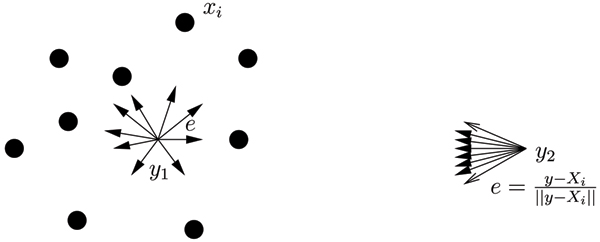
**Illustration of spatial depth function**. *y*_1 _is located deep inside the cloud of *x*'s, summing up *e *will result in a vector with the norm close to 0→
 MathType@MTEF@5@5@+=feaafiart1ev1aaatCvAUfKttLearuWrP9MDH5MBPbIqV92AaeXatLxBI9gBaebbnrfifHhDYfgasaacH8akY=wiFfYdH8Gipec8Eeeu0xXdbba9frFj0=OqFfea0dXdd9vqai=hGuQ8kuc9pgc9s8qqaq=dirpe0xb9q8qiLsFr0=vr0=vr0dc8meaabaqaciaacaGaaeqabaqabeGadaaakeaacuaIWaamgaWcaaaa@2DAC@, since unit vectors have different directions and they cancel each other out. The depth of *y*_1 _is approaching 1. *y*_2 _is outside the data cloud, the sum of *e *has a large norm, thus the depth is approaching 0.

Figure 1 illustrates the spatial depth. Let *e*_*i *_= *S*(*y *-*x*_*i*_) = (*y *- *x*_*i*_) = (||*y *- *x*_*i*_||) where *e*_*i *_represents the unit vector from *y *to *x*_*i*_. When *y *is located deep inside the cloud of *x*'s, summing up *e*_*i *_will result in a vector close to 0→
 MathType@MTEF@5@5@+=feaafiart1ev1aaatCvAUfKttLearuWrP9MDH5MBPbIqV92AaeXatLxBI9gBaebbnrfifHhDYfgasaacH8akY=wiFfYdH8Gipec8Eeeu0xXdbba9frFj0=OqFfea0dXdd9vqai=hGuQ8kuc9pgc9s8qqaq=dirpe0xb9q8qiLsFr0=vr0=vr0dc8meaabaqaciaacaGaaeqabaqabeGadaaakeaacuaIWaamgaWcaaaa@2DAC@, since unit vectors have different directions and they cancel each other out. The depth of *y *is approaching 1. See the diagram on the left in Figure 1. When *y *is outside the data cloud (as in the diagram on the right in Figure 1), the sum of *e*_*i *_has a large norm, thus the depth is approaching 0. The point where the spatial depth attains its maximum value 1 is called the spatial median. The spatial median represents the geometric center of the data, in particular, for a symmetrical distribution, the spatial median is the symmetric center. The spatial depth and the spatial median possess many nice properties. Robustness is one of them.

From the definition of the sample spatial depth, it is not difficult to see that the depth value of a point *x *does not change if any observations are moved to ∞ along the rays connecting them to the point *x*. Thus the spatial depth and the spatial median are highly robust in the presence of outliers. In fact, the breakdown point of the spatial median is 1/2, depending on neither the data nor the dimension and reaching the highest possible value for the translation equivalent location estimator. Here the "breakdown point" is the prevailing quantitative measure of robustness proposed by Donoho and Huber [14]. Roughly speaking, the breakdown point is the minimum fraction of the "bad" data points that can render the estimator beyond any boundary. It is clear to see that one bad point of a data set is enough to ruin the sample mean. Thus, the breakdown point of mean is 1/*n *→ 0, the lowest possible value. That is, the sample mean vector is not robust, hence neither is the clustering method *k*-means which is based on nonrobust sample means.

Unlike the componentwise median, the spatial median is equivariant under orthogonal transformations (e.g. rotations) of the data though it is not equivariant under general affine transformation. The spatial median may not be a reasonable estimate when the scale of different coordinates of the data are widely different. It is, however, very desirable for preprocessed gene data, where variables are isometric.

The complexity of the spatial median is *O*(*n*^2^) for sample size *n *regardless of the dimension. This independence of dimension is particularly important for HDLSS data because high dimension usually causes problems for classical methods.

In our bisecting *k*-spatialMedian algorithm, we propose the use of a robust spatial median to replace the non-robust mean or the less-robust componentwise median to determine the center of the data. The bisecting *k*-spatialMedian algorithm is shown to be more robust than the bisecting *k*-median algorithm in high dimension.

For the selection criterion, we replace the largest variance criterion, which is sensitive to outliers, and propose a depth-based notion, *relative average depth (RAD)*, which characterizes the separatedness of a data set. With its range in [0, 2], a smaller value of the relative average depth indicates less separatedness and a larger value is an indication of higher separatedness. Indeed, in conjunction with the robust spatial median, we can use any existing selection criterion, including largest variance.

## Results and discussion

### Simulation study

**Figure 2:**
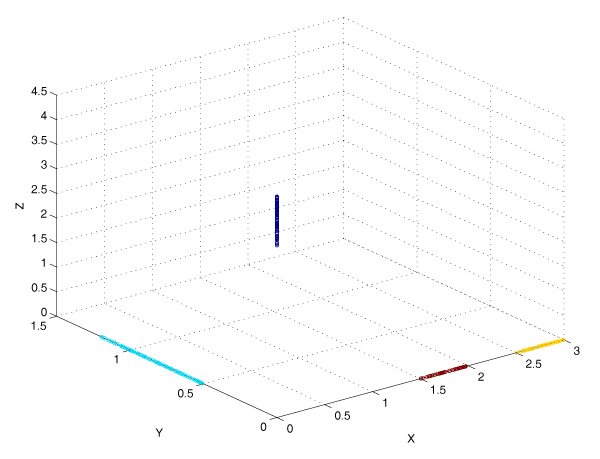
**Simulation to demonstrate the different performance of spatial median and median**. For the four simulated clusters, the bisecting *k*-median failed to identify the four clusters, while the proposed bisecting *k*-spatialMedian successfully separated them, as shown by the four colored group.

To demonstrate the difference in performance between algorithms based on the spatial median and the componentwise median, we conduct a simulation of four clusters in ℝ^3^, see Figure 2. Clusters I and II are comprised of data points (*X*, 0, 0) with *X *generated from the uniform distributions *U*(1.5, 2) and *U*(2.5, 3); and clusters III and IV comprised of data points (0, *Y*, 0) and (0, 0, *Z*) with *Y *from *U*(0.5, 1.2) and *Z *from *U*(3.5, 4.5), where III and IV have the same sample size equaling the sum of the sample sizes of I and II. We observe that the bisecting *k*-median completely fails to separate the four clusters, while the proposed bisecting *k*-spatialMedian successfully finds the four clusters. As shown in Figure 2, the four clusters were perfectly identified by the bisecting *k*-spatialMedian algorithm. Since the output of the bisecting *k*-median is the whole data set, its graph is in one color, without identification of clusters.

The phenomenon observed in the above simulated data seems unrepresentative because the data structure appears so contrived. But actually this is a quite general structure for HDLSS data. In fact, Hall *et al*. [15] show that there is a tendency for HDLSS data to lie deterministically at the vertices of a regular simplex and all the randomness in the data appears as a random rotation of this simplex. Based on this geometric representation, we have shown that the angle between any two distinct data points centered at their common mean is approximately perpendicular, and all these centered data points will lie on the coordinate axes. See the Methods section for more details.

### The bisecting *k*-spatialMedian algorithm

Based on the spatial median, we propose the bisecting *k*-spatialMedian algorithm. Specifically, the bisecting *k*-spatialMedian algorithm recursively splits a cluster by randomly choosing one point *C*_*L *_as the center of one subcluster. Let *C *be the spatial median of the whole data set. Then the center *C*_*R *_of the other subcluster is determined as the symmetric point of *C*_*L *_about *C*, namely, *C*_*R *_= *C *- (*C*_*L *_- *C*). Every point *X *in the cluster is assigned to the subcluster containing *C*_*L *_or *C*_*R *_according to the smaller Euclidean distance ||*X *- *C*_*L*_|| or ||*X *- *C*_*R*_||. This process is repeated until the convergence criterion is met, namely, the centers of the subclusters no longer change. After the cluster is split into two subclusters, a selection rule is needed to determine which subcluster is to be further split.

The basic bisecting *k*-spatialMedian algorithm follows:

INITIALIZE:

   *K*: number of clusters

   *C*: center (spatial median) of the data cluster

   *C*_*L*_: center of left subcluster

   *C*_*R*_: center of right subcluster

FOR *i *= 1 to *K *- 1 do

   choose a cluster to split by the selection rule

   randomly select a point *C*_*L *_as center of left subcluster

   compute *C*_*R *_= *C *- (*C*_*L *_- *C*)

   for *j *= 1 to MAXITER do

      for each data point *X*_*i*_

         if ||*X*_*i *_- *C*_*L*_|| ≥ ||*X*_*i *_- *C*_*R*_||

            assign *X*_*i *_to the right subcluster

         else

            assign *X*_*i *_to the left subcluster

      end

      Let *M*_*L *_be the spatial median of the left subcluster

      Let *M*_*R *_be the spatial median of the right subcluster

      if *M*_*L *_== *C*_*L *_and *M*_*R *_== *C*_*R*_

         break

      *C*_*L *_= *M*_*L*_

      *C*_*R *_= *M*_*R*_

   end

END

### Subcluster selection rule

In the bisecting *k*-spatialMedian algorithm, we need to decide which cluster is to be further split in each step. Selecting the one with the largest variance is a very common approach. Here we propose a new rule based on the statistical spatial depth.

**Figure 3:**
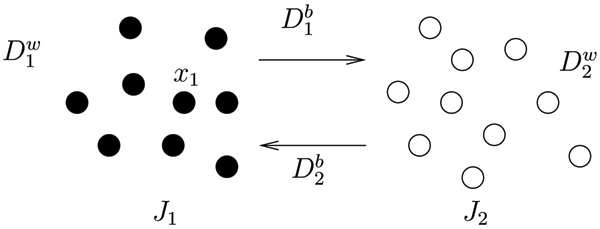
**Graph of within- and between-depth**. The data set is comprised of two clusters *J*_1 _and *J*_2_. D1w
 MathType@MTEF@5@5@+=feaafiart1ev1aaatCvAUfKttLearuWrP9MDH5MBPbIqV92AaeXatLxBI9gBaebbnrfifHhDYfgasaacH8akY=wiFfYdH8Gipec8Eeeu0xXdbba9frFj0=OqFfea0dXdd9vqai=hGuQ8kuc9pgc9s8qqaq=dirpe0xb9q8qiLsFr0=vr0=vr0dc8meaabaqaciaacaGaaeqabaqabeGadaaakeaacqWGebardaqhaaWcbaGaeGymaedabaGaem4DaChaaaaa@3051@ represents the sum of spatial depth of all data points in *J*_1 _with respect to *J*_1_. D2w
 MathType@MTEF@5@5@+=feaafiart1ev1aaatCvAUfKttLearuWrP9MDH5MBPbIqV92AaeXatLxBI9gBaebbnrfifHhDYfgasaacH8akY=wiFfYdH8Gipec8Eeeu0xXdbba9frFj0=OqFfea0dXdd9vqai=hGuQ8kuc9pgc9s8qqaq=dirpe0xb9q8qiLsFr0=vr0=vr0dc8meaabaqaciaacaGaaeqabaqabeGadaaakeaacqWGebardaqhaaWcbaGaeGOmaidabaGaem4DaChaaaaa@3053@ represents the sum of spatial depth of all data points in *J*_2 _with respect to *J*_2_. Note that *D*^*w *^represents "within-depth", because it is the depth of data points with respect to the cluster to which they belong. Let D1b
 MathType@MTEF@5@5@+=feaafiart1ev1aaatCvAUfKttLearuWrP9MDH5MBPbIqV92AaeXatLxBI9gBaebbnrfifHhDYfgasaacH8akY=wiFfYdH8Gipec8Eeeu0xXdbba9frFj0=OqFfea0dXdd9vqai=hGuQ8kuc9pgc9s8qqaq=dirpe0xb9q8qiLsFr0=vr0=vr0dc8meaabaqaciaacaGaaeqabaqabeGadaaakeaacqWGebardaqhaaWcbaGaeGymaedabaGaemOyaigaaaaa@3027@ be the sum of spatial depth of all data points in *J*_1 _with respect to *J*_2_. Similarly, let D2b
 MathType@MTEF@5@5@+=feaafiart1ev1aaatCvAUfKttLearuWrP9MDH5MBPbIqV92AaeXatLxBI9gBaebbnrfifHhDYfgasaacH8akY=wiFfYdH8Gipec8Eeeu0xXdbba9frFj0=OqFfea0dXdd9vqai=hGuQ8kuc9pgc9s8qqaq=dirpe0xb9q8qiLsFr0=vr0=vr0dc8meaabaqaciaacaGaaeqabaqabeGadaaakeaacqWGebardaqhaaWcbaGaeGOmaidabaGaemOyaigaaaaa@3029@ be the sum of spatial depth of all data points in *J*_2 _with respect to *J*_1_. *D*^*b *^represents "between-depth", because they are depth of data points with respect to the other cluster.

Suppose that a data set is naturally composed of two clusters *J*_1 _and *J*_2_. Let D1w
 MathType@MTEF@5@5@+=feaafiart1ev1aaatCvAUfKttLearuWrP9MDH5MBPbIqV92AaeXatLxBI9gBaebbnrfifHhDYfgasaacH8akY=wiFfYdH8Gipec8Eeeu0xXdbba9frFj0=OqFfea0dXdd9vqai=hGuQ8kuc9pgc9s8qqaq=dirpe0xb9q8qiLsFr0=vr0=vr0dc8meaabaqaciaacaGaaeqabaqabeGadaaakeaacqWGebardaqhaaWcbaGaeGymaedabaGaem4DaChaaaaa@3051@ be the sum of spatial depth values of all data points in *J*_1 _with respect to *J*_1_. Let D2w
 MathType@MTEF@5@5@+=feaafiart1ev1aaatCvAUfKttLearuWrP9MDH5MBPbIqV92AaeXatLxBI9gBaebbnrfifHhDYfgasaacH8akY=wiFfYdH8Gipec8Eeeu0xXdbba9frFj0=OqFfea0dXdd9vqai=hGuQ8kuc9pgc9s8qqaq=dirpe0xb9q8qiLsFr0=vr0=vr0dc8meaabaqaciaacaGaaeqabaqabeGadaaakeaacqWGebardaqhaaWcbaGaeGOmaidabaGaem4DaChaaaaa@3053@ be the sum of spatial depth values of all data points in *J*_2 _with respect to *J*_2_. Note that D1w
 MathType@MTEF@5@5@+=feaafiart1ev1aaatCvAUfKttLearuWrP9MDH5MBPbIqV92AaeXatLxBI9gBaebbnrfifHhDYfgasaacH8akY=wiFfYdH8Gipec8Eeeu0xXdbba9frFj0=OqFfea0dXdd9vqai=hGuQ8kuc9pgc9s8qqaq=dirpe0xb9q8qiLsFr0=vr0=vr0dc8meaabaqaciaacaGaaeqabaqabeGadaaakeaacqWGebardaqhaaWcbaGaeGymaedabaGaem4DaChaaaaa@3051@ or D2w
 MathType@MTEF@5@5@+=feaafiart1ev1aaatCvAUfKttLearuWrP9MDH5MBPbIqV92AaeXatLxBI9gBaebbnrfifHhDYfgasaacH8akY=wiFfYdH8Gipec8Eeeu0xXdbba9frFj0=OqFfea0dXdd9vqai=hGuQ8kuc9pgc9s8qqaq=dirpe0xb9q8qiLsFr0=vr0=vr0dc8meaabaqaciaacaGaaeqabaqabeGadaaakeaacqWGebardaqhaaWcbaGaeGOmaidabaGaem4DaChaaaaa@3053@ represents "within-depth", because it is calculated with respect to the cluster to which the data points belong. Let D1b
 MathType@MTEF@5@5@+=feaafiart1ev1aaatCvAUfKttLearuWrP9MDH5MBPbIqV92AaeXatLxBI9gBaebbnrfifHhDYfgasaacH8akY=wiFfYdH8Gipec8Eeeu0xXdbba9frFj0=OqFfea0dXdd9vqai=hGuQ8kuc9pgc9s8qqaq=dirpe0xb9q8qiLsFr0=vr0=vr0dc8meaabaqaciaacaGaaeqabaqabeGadaaakeaacqWGebardaqhaaWcbaGaeGymaedabaGaemOyaigaaaaa@3027@ be the sum of spatial depth values of all data points in *J*_1 _with respect to *J*_2_. Similarly, let D2b
 MathType@MTEF@5@5@+=feaafiart1ev1aaatCvAUfKttLearuWrP9MDH5MBPbIqV92AaeXatLxBI9gBaebbnrfifHhDYfgasaacH8akY=wiFfYdH8Gipec8Eeeu0xXdbba9frFj0=OqFfea0dXdd9vqai=hGuQ8kuc9pgc9s8qqaq=dirpe0xb9q8qiLsFr0=vr0=vr0dc8meaabaqaciaacaGaaeqabaqabeGadaaakeaacqWGebardaqhaaWcbaGaeGOmaidabaGaemOyaigaaaaa@3029@ be the sum of spatial depth values of all data points in *J*_2 _with respect to *J*_1_. D1b
 MathType@MTEF@5@5@+=feaafiart1ev1aaatCvAUfKttLearuWrP9MDH5MBPbIqV92AaeXatLxBI9gBaebbnrfifHhDYfgasaacH8akY=wiFfYdH8Gipec8Eeeu0xXdbba9frFj0=OqFfea0dXdd9vqai=hGuQ8kuc9pgc9s8qqaq=dirpe0xb9q8qiLsFr0=vr0=vr0dc8meaabaqaciaacaGaaeqabaqabeGadaaakeaacqWGebardaqhaaWcbaGaeGymaedabaGaemOyaigaaaaa@3027@ or D2b
 MathType@MTEF@5@5@+=feaafiart1ev1aaatCvAUfKttLearuWrP9MDH5MBPbIqV92AaeXatLxBI9gBaebbnrfifHhDYfgasaacH8akY=wiFfYdH8Gipec8Eeeu0xXdbba9frFj0=OqFfea0dXdd9vqai=hGuQ8kuc9pgc9s8qqaq=dirpe0xb9q8qiLsFr0=vr0=vr0dc8meaabaqaciaacaGaaeqabaqabeGadaaakeaacqWGebardaqhaaWcbaGaeGOmaidabaGaemOyaigaaaaa@3029@ represents ''between-depth", because it is calculated with respect to the other cluster. See Figure 3 for a graphic display. The within-depth is larger when a cluster is more condensed whereas the between-depth is smaller when two clusters are further away from each other.

Let |*J*_1_| and |*J*_2_| represent the number of data points in *J*_1 _and *J*_2 _respectively. The relative average depth is defined as

RAD=D1w|J1|+D2w|J2|−D1b|J1|−D2b|J2|.
 MathType@MTEF@5@5@+=feaafiart1ev1aaatCvAUfKttLearuWrP9MDH5MBPbIqV92AaeXatLxBI9gBaebbnrfifHhDYfgasaacH8akY=wiFfYdH8Gipec8Eeeu0xXdbba9frFj0=OqFfea0dXdd9vqai=hGuQ8kuc9pgc9s8qqaq=dirpe0xb9q8qiLsFr0=vr0=vr0dc8meaabaqaciaacaGaaeqabaqabeGadaaakeaaieaacqWFsbGucqWFbbqqcqWFebarcqGH9aqpdaWcaaqaaiabdseaenaaDaaaleaacqaIXaqmaeaacqWG3bWDaaaakeaacqGG8baFcqWGkbGsdaWgaaWcbaGaeGymaedabeaakiabcYha8baacqGHRaWkdaWcaaqaaiabdseaenaaDaaaleaacqaIYaGmaeaacqWG3bWDaaaakeaacqGG8baFcqWGkbGsdaWgaaWcbaGaeGOmaidabeaakiabcYha8baacqGHsisldaWcaaqaaiabdseaenaaDaaaleaacqaIXaqmaeaacqWGIbGyaaaakeaacqGG8baFcqWGkbGsdaWgaaWcbaGaeGymaedabeaakiabcYha8baacqGHsisldaWcaaqaaiabdseaenaaDaaaleaacqaIYaGmaeaacqWGIbGyaaaakeaacqGG8baFcqWGkbGsdaWgaaWcbaGaeGOmaidabeaakiabcYha8baacqGGUaGlaaa@5854@

As shown from Figure 3, if a data set is naturally composed of two clusters and thus should be split into two, the within-depth should be relatively large and the between-depth relatively small, therefore the relative average depth (RAD) which is essentially the averaged difference between the within-depth and the between-depth will be relatively large compared to the RAD of a data set that is more condensed and cannot be split into two clusters obviously. In fact we have shown that a larger value of RAD indicates less condenseness of a data set. See Section Methods for technical details. Hence we obtain a new selection rule: *A cluster with the largest value of RAD should be selected to split*.

**Figure 4:**
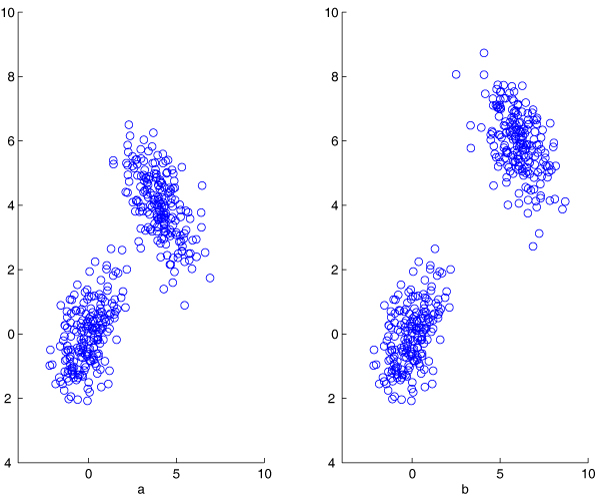
**Simulation to illustrate RAD value**. In Figure a, two clusters are simulated from Normal distribution with mean (0, 0) and (4, 4), respectively. The relative average depth of the data RAD = 0.7864. In Figure b, the mean of the second cluster is moved to (6, 6). The relative average depth RAD = 0.8018.

The following simulation demonstrates the relationship between the value of RAD and the condenseness of a data set. As shown in Figure 4a, two clusters were generated from normal distributions with means *μ*_1 _= (0, 0) and *μ*_2 _= (4, 4), covariances Σ_1 _= (1, 0.5; 0.5, 1) and Σ_2 _= (1, -0.5; -0.5, 1) for the same sample size 200. Obviously the data comprises of two clusters and should be split as such. The relative average depth RAD = 0.7864. If the second cluster is moved from *μ*_2 _= (4, 4) to *μ*_2 _= (6, 6), the two clusters are further away from each other, as shown in Figure 4b. Compared with the previous situation, this new data should have higher priority to be selected for further splitting. The relative average depth increases to RAD = 0.8018. Table 1 lists the values of RAD with one cluster being moved further away from another one with *μ*_1 _= (0, 0). We can see that the RAD value increases slowly when the two clusters are more separated.

**Table 1:** The Relative Average Depth. This table illustrates the relationship of RAD and the separatedness of two clusters. Two clusters are from normal distribution with mean *μ*_1 _= (0, 0)and *μ*_2 _= (2, 2). With *μ*_2 _changing from (2, 2) to (7, 7), the value of RAD increases from 0.6310 to 0.8081 as cluster 2 moves further away from cluster 1.

*μ*_2_	(2,2)	(3,3)	(4,4)	(5,5)	(6,6)	(7,7)
RAD	0.6310	0.7551	0.7864	0.7993	0.8018	0.8081

### Applications

#### Data sets

We use the proposed bisecting *k*-spatialMedian algorithm to analyze two well known data sets. The first is the colon cancer data (Alon data) [16], which is comprised of expression levels of 2000 genes describing 62 samples (40 tumor and 22 normal colon tissues, Affymetrix oligonucleotide arrays). The second is a pediatric Acute Lymphoblastic Leukemia (ALL) data from St. Jude Children's Research Hospital (SJCRH) [17], which includes 12,625 gene expression measurements (Affymetrix arrays) per patient from 246 patients with six different subtypes of ALL.

In the investigation at SJCRH, 246 cases of pediatric ALL were analyzed on the U133 A and B chips, involving six primary subtypes of ALL: BCR-ABL, E2A-PBX1, Hyperdiploid > 50, MLL, T-ALL and TEL. The original data has patient information with two additional subtypes, which did not fit into one of the above primary diagnostic groups or were added for the analysis of relapse and secondary AML. Our study did not include these two subtypes.

#### Design of the experiment

Since the mean is known to lack robustness, we will focus on the comparison of the bisecting algorithms based on componentwise median and spatial median in this paper.

The two data sets were used to compare the performance of the proposed bisecting *k*-spatialMedian with the bisecting *k*-median. Since the class labels of the two data sets are known, the number *K *of clusters is also known. The Alon data set has two classes, so *K *= 2. For the ALL data from SJCRH, *K *= 6. The algorithms are applied on the two datasets and terminated when *K *clusters have been reached.

In order to investigate the performance of the proposed clustering algorithm for HDLSS data, we test them on the two data sets for various dimensions, i.e., different number of genes selected. For the ALL data which has 12265 genes, we test the dimensions D
 MathType@MTEF@5@5@+=feaafiart1ev1aaatCvAUfKttLearuWrP9MDH5MBPbIqV92AaeXatLxBI9gBaebbnrfifHhDYfgasaacH8akY=wiFfYdH8Gipec8Eeeu0xXdbba9frFj0=OqFfea0dXdd9vqai=hGuQ8kuc9pgc9s8qqaq=dirpe0xb9q8qiLsFr0=vr0=vr0dc8meaabaqaciaacaGaaeqabaqabeGadaaakeaat0uy0HwzTfgDPnwy1egaryqtHrhAL1wy0L2yHvdaiqaacqWFdepraaa@3826@ = {100; 200; 500; 1000; 1500; 2000; 3000; 4000; 5000}; for the Alon data which has 2000 genes we test the dimensions D
 MathType@MTEF@5@5@+=feaafiart1ev1aaatCvAUfKttLearuWrP9MDH5MBPbIqV92AaeXatLxBI9gBaebbnrfifHhDYfgasaacH8akY=wiFfYdH8Gipec8Eeeu0xXdbba9frFj0=OqFfea0dXdd9vqai=hGuQ8kuc9pgc9s8qqaq=dirpe0xb9q8qiLsFr0=vr0=vr0dc8meaabaqaciaacaGaaeqabaqabeGadaaakeaat0uy0HwzTfgDPnwy1egaryqtHrhAL1wy0L2yHvdaiqaacqWFdepraaa@3826@ = {50; 100; 200; 500; 1000; 2000}.

For each D
 MathType@MTEF@5@5@+=feaafiart1ev1aaatCvAUfKttLearuWrP9MDH5MBPbIqV92AaeXatLxBI9gBaebbnrfifHhDYfgasaacH8akY=wiFfYdH8Gipec8Eeeu0xXdbba9frFj0=OqFfea0dXdd9vqai=hGuQ8kuc9pgc9s8qqaq=dirpe0xb9q8qiLsFr0=vr0=vr0dc8meaabaqaciaacaGaaeqabaqabeGadaaakeaat0uy0HwzTfgDPnwy1egaryqtHrhAL1wy0L2yHvdaiqaacqWFdepraaa@3826@, we trim the data with only D
 MathType@MTEF@5@5@+=feaafiart1ev1aaatCvAUfKttLearuWrP9MDH5MBPbIqV92AaeXatLxBI9gBaebbnrfifHhDYfgasaacH8akY=wiFfYdH8Gipec8Eeeu0xXdbba9frFj0=OqFfea0dXdd9vqai=hGuQ8kuc9pgc9s8qqaq=dirpe0xb9q8qiLsFr0=vr0=vr0dc8meaabaqaciaacaGaaeqabaqabeGadaaakeaat0uy0HwzTfgDPnwy1egaryqtHrhAL1wy0L2yHvdaiqaacqWFdepraaa@3826@ "most important" genes. We use the SVM-RFE-Annealing algorithm [18] to select the D
 MathType@MTEF@5@5@+=feaafiart1ev1aaatCvAUfKttLearuWrP9MDH5MBPbIqV92AaeXatLxBI9gBaebbnrfifHhDYfgasaacH8akY=wiFfYdH8Gipec8Eeeu0xXdbba9frFj0=OqFfea0dXdd9vqai=hGuQ8kuc9pgc9s8qqaq=dirpe0xb9q8qiLsFr0=vr0=vr0dc8meaabaqaciaacaGaaeqabaqabeGadaaakeaat0uy0HwzTfgDPnwy1egaryqtHrhAL1wy0L2yHvdaiqaacqWFdepraaa@3826@ most important genes. All clustering algorithms are then applied to the trimmed data.

Validation of the clustering results is usually not easy. However, in situations where data are already categorized, as with these data, we can compare the predicted clusters from our algorithms with the true class labels. To display the results, we build a confusion matrix in which rows represent the predicted clusters while columns represent the true clusters. The number in the cell (*i*, *j*) is the number of observations that are from cluster *j *but are predicted to be from cluster *i*. The rows and columns are "matched" by a brute force algorithm, and this is optimistic. Two evaluation measures, Entropy and Misclustering rate, are used. See the Methods section for more details.

Because the bisecting divisive clustering algorithm randomly selects a point as the center of the subcluster *C*_*L*_, it is non-deterministic and therefore yields stochastic clustering results. To evaluate the stochastic clustering result, we ran each algorithm 20 times and calculated the average entropy and misclustering rates as the clustering measures. These algorithms select the next subcluster to split based on the criterion of the largest variance. We compare the performance of our proposed bisecting *k*-spatialMedian with bisecting *k*-median based on the same selection rule, the largest variance, on the two data sets. The performance of bisecting *k*-spatialMedian with the selection criterion of the relative average depth is also presented.

To investigate the robustness of our proposed procedure, we compare the sensitivity of the proposed algorithm to noise with the bisecting *k*-median algorithm. We add noise to the Alon data and then apply the three algorithms (bisecting *k*-median, bisecting *k*-spatialMedian with largest variance splitting rule, bisecting *k*-spatialMedian with RAD splitting rule) on it to investigate their performance.

We generated a percentage of random noise and added to the Alon data by changing the expression value of a point to either the maximum or minimum value of all data points. In this way, some data points are changed to have extreme values and more likely to become outliers. Experiments show that our proposed algorithms based on spatial median perform better than the bisecting *k*-median algorithm in this noisy environment.

#### The result on the Alon data

**Figure 5:**
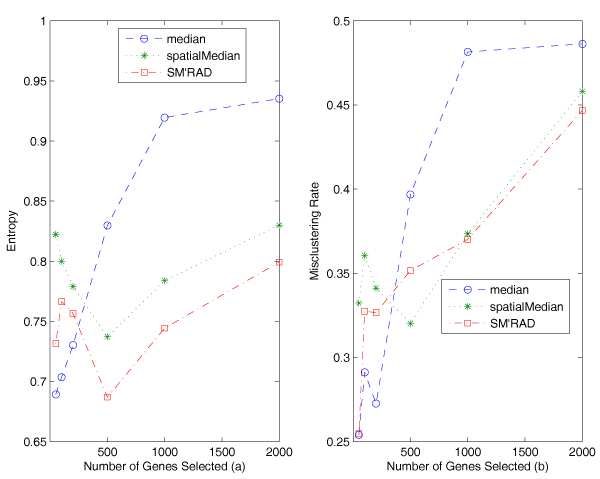
**Experimental results on the Alon data**. Figure a displays comparison of entropy of the clustering algorithms on the trimmed Alon data. Both of the bisecting *k*-spatialMedian algorithms (with the selection criterion relative average depth or the largest variance) outperformed the bisecting *k*-median algorithm. Figure b displays comparison of misclustering rates of the clustering algorithms on the trimmed Alon data. Both of the bisecting *k*-spatialMedian algorithms (with the selection criterion relative average depth or the largest variance) outperformed the bisecting *k*-median algorithm.

Figure 5 reports the entropy and the misclustering rates of the algorithms on the trimmed Alon data. These algorithms are the bisecting *k*-median (median), the bisecting *k*-spatialMedian (spatialMedian), the bisecting *k*-spatialMedian based on the selection criterion of the relative average depth (SM-RAD). The first two algorithms use the largest variance as selection rule.

From Figure 5a and 5b, we can see that both of the algorithms using spatial median have lower entropy and misclustering rates than the one using componentwise median in most of the cases. When we use more than 400 genes in clustering, the algorithms using spatial median are better than the one using componentwise median, which demonstrates that spatial median is more robust in higher dimensional data. Also the performance of the algorithm using median is decreasing dramatically with dimensions increasing from 200 to 1000, while the performance of the algorithms using spatial median does not degrade as much.

**Figure 6:**
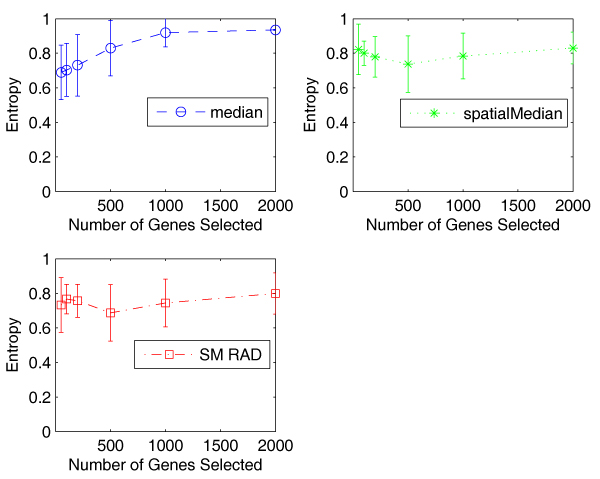
**Comparison of the entropy values with standard deviation of the three algorithms on the Alon data**. The error bars show that the three algorithms have similar standard deviation in calculating entropy values.

Figure 6 shows the entropy values with standard deviation of the three algorithms. We can see that the three algorithms display similar variation, about 0.2 in most cases. The very similar results are obtained by using misclustering rate.

Additional file 1 gives an example of the relationship of the number of runs and average entropy of the Alon data. In additional file 1, the entropy values get more stable with the number of runs increasing, which justifies the need of running the clustering algorithms multiple times. The average misclustering rate and the number of runs have the similar relationship.

#### The result on the SJCRH data

**Figure 7:**
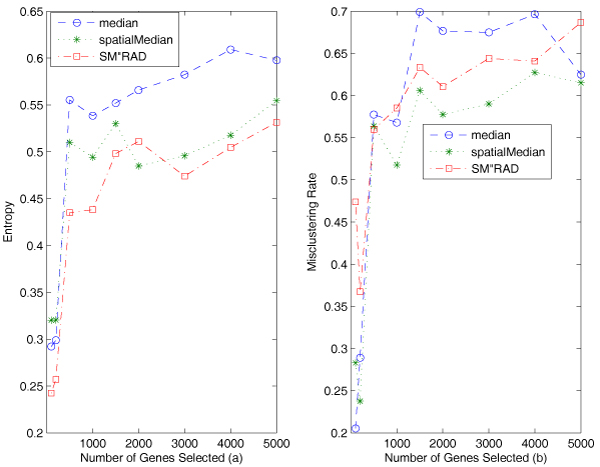
**Experimental results on the SJCRH data**. Figure a displays comparison of entropy of the clustering algorithms on the trimmed SJCRH data. Both spatial median algorithms are superior to the median algorithm. Figure b displays comparison of misclustering rates of the algorithms on the SJCRH data. Both spatial median algorithms are superior to the median algorithm.

Similarly, Figure 7 reports the entropy and misclustering rates of the algorithms on the trimmed SJCRH data. We can see that in most of the cases after 500 genes are used, both of the algorithms using spatial median are better than the bisecting *k*-median. The largest difference between bisecting *k*-spatialMedian and median is more than 10%. The results are consistent with the results on the Alon data.

**Figure 8:**
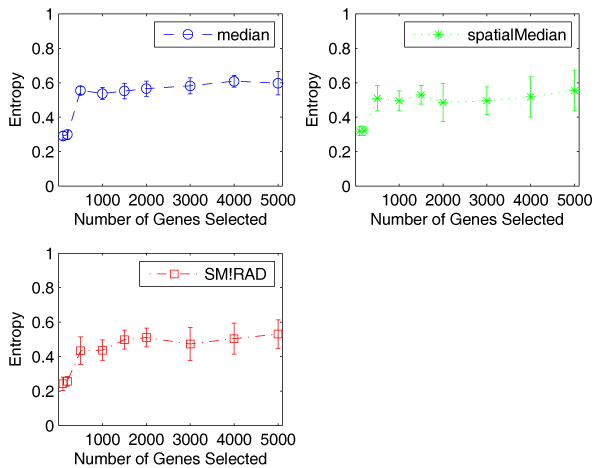
**Comparison of the entropy values with standard deviation of the three algorithms on the SJCRH data**. The error bars show that the three algorithms have similar standard deviation in calculating entropy values.

Similarly, Figure 8 shows the entropy values with standard deviation of the three algorithms. We can see that the three algorithms display similar variation, less than 0.1 in most cases, although the algorithm using median achieves the lowest standard deviation. Standard deviation appears to be more consistent with median than with spatialMedian on the SJCRH data. The very similar results are obtained by using misclustering rate.

Additional file 2 gives an example of the relationship of the number of runs and average entropy of the SJCRH data. In additional file 2, the entropy values get more stable with the number of runs increasing. The average misclustering rate and the number of runs have the similar relationship.

#### The result on the noisy Alon data

We randomly add noise to the Alon data to see how well the algorithms based on the componentwise median and the spatial median perform in a noisy environment.

To this end, we randomly pick 10% of data from the Alon data, and reset their values to be either the maximum or minimum value in the data matrix.

We applied the three algorithms to this noisy data and observed that all the algorithms have been influenced by the noise. However, the bisecting *k*-median is more susceptible to the noise, which can be demonstrated by the fact that it cannot separate the two clusters at all.

This process is repeated several times and the results are very consistent. We further increase the amount of noise from 10% to 20% and get a similar result.

**Figure 9:**
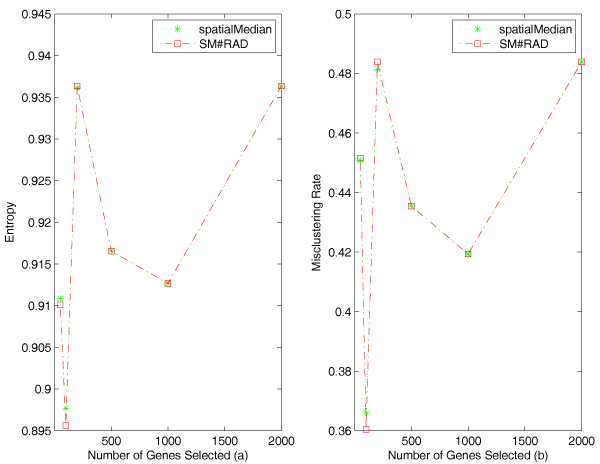
**Experimental results on the noisy Alon data**. Figure a displays comparison of entropy of the clustering algorithms on the noisy Alon data. The performance of the bisecting *k*-spatialMedian algorithms (with the selection criterion relative average depth or the largest variance) are very similar. The bisecting *k*-median algorithm cannot separate the two clusters, so its entropy value is not available thus not shown in this figure. Figure b displays comparison of misclustering rates of the clustering algorithms on the noisy Alon data. The performance of the bisecting *k*-spatialMedian algorithms (with the selection criterion relative average depth or the largest variance) are very similar. The bisecting *k*-median algorithm cannot separate the two clusters, so its misclustering rate is not available thus not shown in this figure.

Figure 9 shows that the algorithms based on spatial median have very similar entropy values and mis-clustering rates on the noisy Alon data. Since the bisecting *k*-median cannot separate the two clusters, its entropy value or misclustring rate is not available thus not shown in Figure 9.

## Conclusion

The spatial depth function provides a robust location estimator whereas componentwise median may not work well in high dimension and low sample size data, which is illustrated by easily designed simulation. The experimental results on real data sets further verify that the spatial median based bisecting clustering algorithm is more robust to outliers and noise in high dimensional data, such as gene expression data, than the bisecting *k*-median algorithm.

## Methods

### Geometric structure of HDLSS data

In their 2005 article, Hall, Marron and Neeman [15] point out that for *d*-dimensional i.i.d. random vectors *Z*_1_,...,*Z*_*m *_whose coordinates are i.i.d. with the standard normal N
 MathType@MTEF@5@5@+=feaafiart1ev1aaatCvAUfKttLearuWrP9MDH5MBPbIqV92AaeXatLxBI9gBaebbnrfifHhDYfgasaacH8akY=wiFfYdH8Gipec8Eeeu0xXdbba9frFj0=OqFfea0dXdd9vqai=hGuQ8kuc9pgc9s8qqaq=dirpe0xb9q8qiLsFr0=vr0=vr0dc8meaabaqaciaacaGaaeqabaqabeGadaaakeaat0uy0HwzTfgDPnwy1egaryqtHrhAL1wy0L2yHvdaiqaacqWFneVtaaa@383A@(0, 1), all distinct pairwise Euclidean distances ||*Z*_*i *_- *Z*_*j*_||_*d *_are approximately equal and all pairwise angles ang(*Z*_*i*_, *Z*_*j*_) are approximately perpendicular for large *d*. Without normality assumptions they further demonstrate that all pairwise distances are still approximately equal under certain moment assumptions. Specially they give the following geometric representation. For an infinite sequence *X *= (*X*^(1)^, *X*^(2)^,...) of random variables, assume

(i) There exists a constant M such that E
 MathType@MTEF@5@5@+=feaafiart1ev1aaatCvAUfKttLearuWrP9MDH5MBPbIqV92AaeXatLxBI9gBaebbnrfifHhDYfgasaacH8akY=wiFfYdH8Gipec8Eeeu0xXdbba9frFj0=OqFfea0dXdd9vqai=hGuQ8kuc9pgc9s8qqaq=dirpe0xb9q8qiLsFr0=vr0=vr0dc8meaabaqaciaacaGaaeqabaqabeGadaaakeaatuuDJXwAK1uy0HMmaeHbfv3ySLgzG0uy0HgiuD3BaGabaiab=ri8fbaa@388C@|*X*^(*i*)^|^4 ^<*M *for all *i *= 1, 2,....

(ii) There is a constant *σ*^2 ^such that

1d∑k=1dVar(X(k))→σ2,d→∞.     (1)
 MathType@MTEF@5@5@+=feaafiart1ev1aaatCvAUfKttLearuWrP9MDH5MBPbIqV92AaeXatLxBI9gBaebbnrfifHhDYfgasaacH8akY=wiFfYdH8Gipec8Eeeu0xXdbba9frFj0=OqFfea0dXdd9vqai=hGuQ8kuc9pgc9s8qqaq=dirpe0xb9q8qiLsFr0=vr0=vr0dc8meaabaqaciaacaGaaeqabaqabeGadaaakeaafaqabeqacaaabaWaaSaaaeaacqaIXaqmaeaacqWGKbazaaWaaabCaeaatuuDJXwAK1uy0HMmaeHbfv3ySLgzG0uy0HgiuD3BaGabaiab=vj8wHqaaiab+fgaHjab+jhaYjabcIcaOiabdIfaynaaCaaaleqabaGaeiikaGIaem4AaSMaeiykaKcaaOGaeiykaKcaleaacqWGRbWAcqGH9aqpcqaIXaqmaeaacqWGKbaza0GaeyyeIuoakiabgkziUIGaciab9n8aZnaaCaaaleqabaGaeGOmaidaaOGaeiilaWcabaGaemizaqMaeyOKH4QaeyOhIuQaeiOla4caaaaa@5639@

(iii) The infinite sequence *X *is *ρ *mixing, for detail, see [15].

Let *X*(*d*) = (*X*^(1)^,...,*X*^(*d*)^) be a coordinate projection of *X *into the *d*-dimensional space ℝ^*d *^and let *X*_1_(*d*),...,*X*_*m*_(*d*) be independent and identical copies of *X*(*d*). Then for all distinct pairs *X*_*i *_≠ *X*_*j*_, the distances ‖Xi−Xj‖d=(∑k=1d(Xi(k)−Xj(k))2)1/2
 MathType@MTEF@5@5@+=feaafiart1ev1aaatCvAUfKttLearuWrP9MDH5MBPbIqV92AaeXatLxBI9gBaebbnrfifHhDYfgasaacH8akY=wiFfYdH8Gipec8Eeeu0xXdbba9frFj0=OqFfea0dXdd9vqai=hGuQ8kuc9pgc9s8qqaq=dirpe0xb9q8qiLsFr0=vr0=vr0dc8meaabaqaciaacaGaaeqabaqabeGadaaakeaadaqbdaqaaiabdIfaynaaBaaaleaacqWGPbqAaeqaaOGaeyOeI0IaemiwaG1aaSbaaSqaaiabdQgaQbqabaaakiaawMa7caGLkWoadaWgaaWcbaGaemizaqgabeaakiabg2da9maabmaabaWaaabmaeaacqGGOaakcqWGybawdaqhaaWcbaGaemyAaKgabaGaeiikaGIaem4AaSMaeiykaKcaaOGaeyOeI0IaemiwaG1aa0baaSqaaiabdQgaQbqaaiabcIcaOiabdUgaRjabcMcaPaaakiabcMcaPaWcbaGaem4AaSMaeyypa0JaeGymaedabaGaemizaqganiabggHiLdGcdaahaaWcbeqaaiabikdaYaaaaOGaayjkaiaawMcaamaaCaaaleqabaGaeGymaeJaei4la8IaeGOmaidaaaaa@5395@ are approximately equal:

*d*^-1/2^||*X*_*i *_- *X*_*j*_||_*d *_→ 2
                  MathType@MTEF@5@5@+=feaafiart1ev1aaatCvAUfKttLearuWrP9MDH5MBPbIqV92AaeXatLxBI9gBaebbnrfifHhDYfgasaacH8akY=wiFfYdH8Gipec8Eeeu0xXdbba9frFj0=OqFfea0dXdd9vqai=hGuQ8kuc9pgc9s8qqaq=dirpe0xb9q8qiLsFr0=vr0=vr0dc8meaabaqaciaacaGaaeqabaqabeGadaaakeaadaGcaaqaaiabikdaYaWcbeaaaaa@2DB9@*σ*, *d *→ ∞.     (2)

Observing their result, we find, with *μ *= E
 MathType@MTEF@5@5@+=feaafiart1ev1aaatCvAUfKttLearuWrP9MDH5MBPbIqV92AaeXatLxBI9gBaebbnrfifHhDYfgasaacH8akY=wiFfYdH8Gipec8Eeeu0xXdbba9frFj0=OqFfea0dXdd9vqai=hGuQ8kuc9pgc9s8qqaq=dirpe0xb9q8qiLsFr0=vr0=vr0dc8meaabaqaciaacaGaaeqabaqabeGadaaakeaatuuDJXwAK1uy0HMmaeHbfv3ySLgzG0uy0HgiuD3BaGabaiab=ri8fbaa@388C@*X*_*i*_, that

*d*^-1/2^||*X*_*i *_- *X*_*j*_||_*d *_- *d*^-1/2^||*X*_*i *_- *μ*||_*d *_- *d*^-1/2^||*X*_*j *_- *μ*||_*d *_→ 0,

as *d *→ ∞. This shows, in view of the Pythagorean theorem, the following fact.

**Fact 1. **Under the above assumptions (i)–(iii), the pairwise angle between distinct *X*_*i *_- *μ*_*i *_and *X*_*j *_- *μ*_*j *_is approximately perpendicular:

ang(*X*_*i *_- *μ*, *X*_*j *_- *μ*) = *π*/2 + *O*_*p*_(*d*^-1/2^).     (3)

It is well known that spatial depth function attains its maximum value at the symmetric center of a distribution under very mild assumptions and the spatial median is the maximizer. Thus the spatial median is the center of the regular simplex when the number of observations at every vertex is equal.

This exhibits that, for HDLSS data, the spatial depth can find the center and this helps find the right clusters, while a componentwise median may fail to find the symmetric center and thus the componentwise-median-based procedures may be unable to find the right clusters. In fact, we expanded the dimension of our data set from the previous simulation which has three dimensions as shown in Figure 2 and found that the componentwise-median-based bisecting *k*-median breaks down more easily with increasing dimension while the bisecting *k*-spatialMedian does not.

### Theoretical verification of subcluster selection rule

Suppose that we have collected observations *X*_*j *_: *j *∈ *J *= {1,...,*n*} which are points in ℝ^*d*^. Suppose also that these observations are from two sources. We want to find a rule to measure the condenseness of the data, in other words, how different the two resources are. Statistically we suppose that *X*_*j *_: *j *∈ *J *= {1,...,*n*} are independent observations from a population distribution *F*. Suppose that *X*_*j *_: *j *∈ *J*_1 _and *X*_*j *_: *j *∈ *J*_2 _are from population distributions *F*_1 _and *F*_2 _respectively with *J*_1_, *J*_2 _being partitions of *J*. For convenience we refer to these two subclusters of *J *as *J*_1 _and *J*_2 _respectively. We want to use the robust depth functions to measure the condenseness of *J*, or in other words, the separatedness of *J*_1 _and *J*_2_. Let *D*(*x*, *F*) be the population depth of a point *x *with respect to *F*. The sample depth is *D*(*x*, *J*) ≡ *D*(*x*, *F*_*n*_) where *F*_*n *_is the empirical distribution of *F*.

One of the desirable properties for most of the depth functions is monotonicity relative to the deepest point, i.e., the depth-based multivariate median. Specifically, as a point *x *∈ ℝ^*d *^moves away from the multivariate median *M *along any fixed ray through *M*, the depth at *x *decreases monotonically, namely,

*D*(*x*, *F*) ≤ *D*(*M *+ *α*(*x *- *M*), *F*), *x *∈ ℝ^*d*^     (4)

holds for all *α *∈ [0, 1]. This property can be used to characterize the separatedness of the two clusters. For unambiguity let us write *X*_*i *_for the observations *X*_*i *_: *i *∈ *J*_1 _and *Y*_*j *_for *X*_*j *_: *j *∈ *J*_2_.

Suppose that clusters *J*_1 _and *J*_2 _are separated. Observe that, by the monotonicity (4), if *X*_*i *_is from cluster *J*_1 _and *Y*_*j *_from cluster *J*_2 _then the depth of *X*_*i *_should be larger than the depth of *Y*_*j*_, both with respect to cluster *J*_1_. Namely,

*D*(*X*_*i*_, *J*_1_) ≽ *D*(*Y*_*j*_, *J*_1_), *i *∈ *J*_1_, *j *∈ *J*_2_,     (5)

where ≽ is the stochastic ordering in the sense that *η *≽ *ξ*, if and only if ℙ (*η *≥ *ξ*) ≥ 1/2 for two random variables *η*, *ξ*. The inequalities are useful in characterizing the separatedness of two clusters *J*_1 _and *J*_2_.

Note that *D*(*X*_*i*_, *J*_1_) and *D*(*Y*_*j*_, *J*_1_) are called within- and between-depth by [19] and [2]. The population version of (5) is

*D*(*X*, *F*_1_) ≽ *D*(*Y*, *F*_1_), *X *~ *F*_1_, *Y *~ *F*_2_.     (6)

The inequality has clear geometric interpretation. With respect to distribution *F*_1_, the depth of random variable *X *from distribution *F*_1 _is larger than the depth of random variable *Y *from distribution *F*_2_. Indeed we have the following fact for the spatial depth.

**Fact 2. **Suppose *F*_2 _= *F*_1_(· - *c*) where *c *∈ ℝ^*d *^is a constant vector. If *F*_1 _has finite support, then for *X *~ *F*_1 _and *Y *~ *F*_2_,

lim⁡‖c‖→∞ℙ(SPD(X,F1)≥SPD(Y,F1))=1.
 MathType@MTEF@5@5@+=feaafiart1ev1aaatCvAUfKttLearuWrP9MDH5MBPbIqV92AaeXatLxBI9gBaebbnrfifHhDYfgasaacH8akY=wiFfYdH8Gipec8Eeeu0xXdbba9frFj0=OqFfea0dXdd9vqai=hGuQ8kuc9pgc9s8qqaq=dirpe0xb9q8qiLsFr0=vr0=vr0dc8meaabaqaciaacaGaaeqabaqabeGadaaakeaadaWfqaqaaiGbcYgaSjabcMgaPjabc2gaTbWcbaWaauWaaeaacqWGJbWyaiaawMa7caGLkWoacqGHsgIRcqGHEisPaeqaamrr1ngBPrwtHrhAYaqeguuDJXwAKbstHrhAGq1DVbaceaGccqWFzecucqGGOaakieaacqGFtbWucqGFqbaucqGFebarcqGGOaakcqWGybawcqGGSaalcqWGgbGrdaWgaaWcbaGaeGymaedabeaakiabcMcaPiabgwMiZkab+nfatjab+bfaqjab+reaejabcIcaOiabdMfazjabcYcaSiabdAeagnaaBaaaleaacqaIXaqmaeqaaOGaeiykaKIaeiykaKIaeyypa0JaeGymaeJaeiOla4caaa@5CAF@

Proof. Using ||E
 MathType@MTEF@5@5@+=feaafiart1ev1aaatCvAUfKttLearuWrP9MDH5MBPbIqV92AaeXatLxBI9gBaebbnrfifHhDYfgasaacH8akY=wiFfYdH8Gipec8Eeeu0xXdbba9frFj0=OqFfea0dXdd9vqai=hGuQ8kuc9pgc9s8qqaq=dirpe0xb9q8qiLsFr0=vr0=vr0dc8meaabaqaciaacaGaaeqabaqabeGadaaakeaatuuDJXwAK1uy0HMmaeHbfv3ySLgzG0uy0HgiuD3BaGabaiab=ri8fbaa@388C@*S*(*x *- *ξ*)||^2 ^= E
 MathType@MTEF@5@5@+=feaafiart1ev1aaatCvAUfKttLearuWrP9MDH5MBPbIqV92AaeXatLxBI9gBaebbnrfifHhDYfgasaacH8akY=wiFfYdH8Gipec8Eeeu0xXdbba9frFj0=OqFfea0dXdd9vqai=hGuQ8kuc9pgc9s8qqaq=dirpe0xb9q8qiLsFr0=vr0=vr0dc8meaabaqaciaacaGaaeqabaqabeGadaaakeaatuuDJXwAK1uy0HMmaeHbfv3ySLgzG0uy0HgiuD3BaGabaiab=ri8fbaa@388C@_*ξ*,*η*_*S*^⊥^(*x *- *ξ*)*S*(*x *- *η*) where *ξ*, *η *are independent and have a common distribution and E
 MathType@MTEF@5@5@+=feaafiart1ev1aaatCvAUfKttLearuWrP9MDH5MBPbIqV92AaeXatLxBI9gBaebbnrfifHhDYfgasaacH8akY=wiFfYdH8Gipec8Eeeu0xXdbba9frFj0=OqFfea0dXdd9vqai=hGuQ8kuc9pgc9s8qqaq=dirpe0xb9q8qiLsFr0=vr0=vr0dc8meaabaqaciaacaGaaeqabaqabeGadaaakeaatuuDJXwAK1uy0HMmaeHbfv3ySLgzG0uy0HgiuD3BaGabaiab=ri8fbaa@388C@_*ξ*,*η *_is calculated under the joint probability of *ξ *and *η*, one has

ℙ(SPD(*X*, *F*_1_) ≥ SPD(*Y*, *F*_1_)) = ℙ(E
 MathType@MTEF@5@5@+=feaafiart1ev1aaatCvAUfKttLearuWrP9MDH5MBPbIqV92AaeXatLxBI9gBaebbnrfifHhDYfgasaacH8akY=wiFfYdH8Gipec8Eeeu0xXdbba9frFj0=OqFfea0dXdd9vqai=hGuQ8kuc9pgc9s8qqaq=dirpe0xb9q8qiLsFr0=vr0=vr0dc8meaabaqaciaacaGaaeqabaqabeGadaaakeaatuuDJXwAK1uy0HMmaeHbfv3ySLgzG0uy0HgiuD3BaGabaiab=ri8fbaa@388C@_*ξ*,*η*_[*S*^⊥^(*X *- *ξ*)*S*(*X *- *η*) - *S*^⊥^(*X *- *ξ *+ *c*)*S*(*X *- *η *+ *c*)] ≤ 0).

It is easy to see *S*^⊥^(*X *- *ξ *+ *c*)*S*(*X *- *η *+ *c*) → 1 as ||*c*|| → ∞. Combining the above yields the desired result and the proof is complete.

Fact 2 implies that if one cluster is shifted away further enough then we have the stochastic ordering (6) and hence (5) for large sample.

However, the inequality is a little too strong. Instead of (6) holding for all *X *~ *F*_1 _and *Y *~ *F*_2_, a less restrictive inequality would be to require (6) to hold on average, i.e.,

EF1D(X,F1)≥EF2D(Y,F1).     (7)
 MathType@MTEF@5@5@+=feaafiart1ev1aaatCvAUfKttLearuWrP9MDH5MBPbIqV92AaeXatLxBI9gBaebbnrfifHhDYfgasaacH8akY=wiFfYdH8Gipec8Eeeu0xXdbba9frFj0=OqFfea0dXdd9vqai=hGuQ8kuc9pgc9s8qqaq=dirpe0xb9q8qiLsFr0=vr0=vr0dc8meaabaqaciaacaGaaeqabaqabeGadaaakeaatuuDJXwAK1uy0HMmaeHbfv3ySLgzG0uy0HgiuD3BaGabaiab=ri8fnaaBaaaleaacqWGgbGrdaWgaaadbaGaeGymaedabeaaaSqabaGccqWGebarcqGGOaakcqWGybawcqGGSaalcqWGgbGrdaWgaaWcbaGaeGymaedabeaakiabcMcaPiabgwMiZkab=ri8fnaaBaaaleaacqWGgbGrdaWgaaadbaGaeGOmaidabeaaaSqabaGccqWGebarcqGGOaakcqWGzbqwcqGGSaalcqWGgbGrdaWgaaWcbaGaeGymaedabeaakiabcMcaPiabc6caUaaa@508C@

Analogously,

EF2D(Y,F2)≥EF1D(X,F2).     (8)
 MathType@MTEF@5@5@+=feaafiart1ev1aaatCvAUfKttLearuWrP9MDH5MBPbIqV92AaeXatLxBI9gBaebbnrfifHhDYfgasaacH8akY=wiFfYdH8Gipec8Eeeu0xXdbba9frFj0=OqFfea0dXdd9vqai=hGuQ8kuc9pgc9s8qqaq=dirpe0xb9q8qiLsFr0=vr0=vr0dc8meaabaqaciaacaGaaeqabaqabeGadaaakeaatuuDJXwAK1uy0HMmaeHbfv3ySLgzG0uy0HgiuD3BaGabaiab=ri8fnaaBaaaleaacqWGgbGrdaWgaaadbaGaeGOmaidabeaaaSqabaGccqWGebarcqGGOaakcqWGzbqwcqGGSaalcqWGgbGrdaWgaaWcbaGaeGOmaidabeaakiabcMcaPiabgwMiZkab=ri8fnaaBaaaleaacqWGgbGrdaWgaaadbaGaeGymaedabeaaaSqabaGccqWGebarcqGGOaakcqWGybawcqGGSaalcqWGgbGrdaWgaaWcbaGaeGOmaidabeaakiabcMcaPiabc6caUaaa@5090@

Indeed similarly to the proof of Fact 2, we may establish the above two inequalities which shall be discussed elsewhere.

In order to characterize the separatedness of the two clusters we first introduce the following notions.

#### Depth total, Within- and Between-Depth

Let *D*_|*J*| _be the sum of the sample depths of all observations on *J*, i.e., *D*_|*J*| _= ∑_*j*∈*J*_*D*(*X*_*j*_, *J*), and we call it the depth total on *J*. We call the depth total on *J*_1 _and *J*_2_,

D1w≡∑i∈J1D(Xi,J1),D2w≡∑j∈J2D(Yj,J2),
 MathType@MTEF@5@5@+=feaafiart1ev1aaatCvAUfKttLearuWrP9MDH5MBPbIqV92AaeXatLxBI9gBamXvP5wqSXMqHnxAJn0BKvguHDwzZbqegyvzYrwyUfgarqqtubsr4rNCHbGeaGqiA8vkIkVAFgIELiFeLkFeLk=iY=Hhbbf9v8qqaqFr0xc9pk0xbba9q8WqFfeaY=biLkVcLq=JHqVepeea0=as0db9vqpepesP0xe9Fve9Fve9GapdbaqaaeGacaGaaiaabeqaamqadiabaaGcbaqbaeqabeGaaaqaaiabdseaenaaDaaaleaacqaIXaqmaeaacqWG3bWDaaGccqGHHjIUdaaeqbqaaiabdseaejabcIcaOiabdIfaynaaBaaaleaacqWGPbqAaeqaaOGaeiilaWIaemOsaO0aaSbaaSqaaiabigdaXaqabaGccqGGPaqkcqGGSaalaSqaaiabdMgaPjabgIGiolabdQeaknaaBaaameaacqaIXaqmaeqaaaWcbeqdcqGHris5aaGcbaGaemiraq0aa0baaSqaaiabikdaYaqaaiabdEha3baakiabggMi6oaaqafabaGaemiraqKaeiikaGIaemywaK1aaSbaaSqaaiabdQgaQbqabaGccqGGSaalcqWGkbGsdaWgaaWcbaGaeGOmaidabeaakiabcMcaPiabcYcaSaWcbaGaemOAaOMaeyicI4SaemOsaO0aaSbaaWqaaiabikdaYaqabaaaleqaniabggHiLdaaaaaa@69AC@

the within-depth, and

D1b=∑i∈J1D(Xi,J2),D2b=∑j∈J2D(Yj,J1),
 MathType@MTEF@5@5@+=feaafiart1ev1aaatCvAUfKttLearuWrP9MDH5MBPbIqV92AaeXatLxBI9gBaebbnrfifHhDYfgasaacH8akY=wiFfYdH8Gipec8Eeeu0xXdbba9frFj0=OqFfea0dXdd9vqai=hGuQ8kuc9pgc9s8qqaq=dirpe0xb9q8qiLsFr0=vr0=vr0dc8meaabaqaciaacaGaaeqabaqabeGadaaakeaafaqabeqacaaabaGaemiraq0aa0baaSqaaiabigdaXaqaaiabdkgaIbaakiabg2da9maaqafabaGaemiraqKaeiikaGIaemiwaG1aaSbaaSqaaiabdMgaPbqabaGccqGGSaalcqWGkbGsdaWgaaWcbaGaeGOmaidabeaakiabcMcaPiabcYcaSaWcbaGaemyAaKMaeyicI4SaemOsaO0aaSbaaWqaaiabigdaXaqabaaaleqaniabggHiLdaakeaacqWGebardaqhaaWcbaGaeGOmaidabaGaemOyaigaaOGaeyypa0ZaaabuaeaacqWGebarcqGGOaakcqWGzbqwdaWgaaWcbaGaemOAaOgabeaakiabcYcaSiabdQeaknaaBaaaleaacqaIXaqmaeqaaOGaeiykaKIaeiilaWcaleaacqWGQbGAcqGHiiIZcqWGkbGsdaWgaaadbaGaeGOmaidabeaaaSqab0GaeyyeIuoaaaaaaa@5793@

the between-depth. Figure 3 is a graphic display of these notations.

Summing up *i *∈ *J*_1_, *j *∈ *J*_2 _through (5) yields

D1w|J1|≥D2b|J2|.     (9)
 MathType@MTEF@5@5@+=feaafiart1ev1aaatCvAUfKttLearuWrP9MDH5MBPbIqV92AaeXatLxBI9gBaebbnrfifHhDYfgasaacH8akY=wiFfYdH8Gipec8Eeeu0xXdbba9frFj0=OqFfea0dXdd9vqai=hGuQ8kuc9pgc9s8qqaq=dirpe0xb9q8qiLsFr0=vr0=vr0dc8meaabaqaciaacaGaaeqabaqabeGadaaakeaadaWcaaqaaiabdseaenaaDaaaleaacqaIXaqmaeaacqWG3bWDaaaakeaacqGG8baFcqWGkbGsdaWgaaWcbaGaeGymaedabeaakiabcYha8baacqGHLjYSdaWcaaqaaiabdseaenaaDaaaleaacqaIYaGmaeaacqWGIbGyaaaakeaacqGG8baFcqWGkbGsdaWgaaWcbaGaeGOmaidabeaakiabcYha8baacqGGUaGlaaa@4134@

Analogously,

D2w|J2|≥D1b|J1|.     (10)
 MathType@MTEF@5@5@+=feaafiart1ev1aaatCvAUfKttLearuWrP9MDH5MBPbIqV92AaeXatLxBI9gBaebbnrfifHhDYfgasaacH8akY=wiFfYdH8Gipec8Eeeu0xXdbba9frFj0=OqFfea0dXdd9vqai=hGuQ8kuc9pgc9s8qqaq=dirpe0xb9q8qiLsFr0=vr0=vr0dc8meaabaqaciaacaGaaeqabaqabeGadaaakeaadaWcaaqaaiabdseaenaaDaaaleaacqaIYaGmaeaacqWG3bWDaaaakeaacqGG8baFcqWGkbGsdaWgaaWcbaGaeGOmaidabeaakiabcYha8baacqGHLjYSdaWcaaqaaiabdseaenaaDaaaleaacqaIXaqmaeaacqWGIbGyaaaakeaacqGG8baFcqWGkbGsdaWgaaWcbaGaeGymaedabeaakiabcYha8baacqGGUaGlaaa@4134@

These two inequalities can be used to characterize the separatedness of two clusters *J*_1 _and *J*_2_. To exploit the inequalities simultaneously we introduce the following.

#### Relative average depth

RAD=D1w|J1|+D2w|J2|−D1b|J1|−D2b|J2|.     (11)
 MathType@MTEF@5@5@+=feaafiart1ev1aaatCvAUfKttLearuWrP9MDH5MBPbIqV92AaeXatLxBI9gBaebbnrfifHhDYfgasaacH8akY=wiFfYdH8Gipec8Eeeu0xXdbba9frFj0=OqFfea0dXdd9vqai=hGuQ8kuc9pgc9s8qqaq=dirpe0xb9q8qiLsFr0=vr0=vr0dc8meaabaqaciaacaGaaeqabaqabeGadaaakeaaieaacqWFsbGucqWFbbqqcqWFebarcqGH9aqpdaWcaaqaaiabdseaenaaDaaaleaacqaIXaqmaeaacqWG3bWDaaaakeaacqGG8baFcqWGkbGsdaWgaaWcbaGaeGymaedabeaakiabcYha8baacqGHRaWkdaWcaaqaaiabdseaenaaDaaaleaacqaIYaGmaeaacqWG3bWDaaaakeaacqGG8baFcqWGkbGsdaWgaaWcbaGaeGOmaidabeaakiabcYha8baacqGHsisldaWcaaqaaiabdseaenaaDaaaleaacqaIXaqmaeaacqWGIbGyaaaakeaacqGG8baFcqWGkbGsdaWgaaWcbaGaeGymaedabeaakiabcYha8baacqGHsisldaWcaaqaaiabdseaenaaDaaaleaacqaIYaGmaeaacqWGIbGyaaaakeaacqGG8baFcqWGkbGsdaWgaaWcbaGaeGOmaidabeaakiabcYha8baacqGGUaGlaaa@5854@

is called the *relative average depth*. If clusters *F*_1 _and *F*_2 _are separated, then the two inequalities (7) and (8) should hold. We believe that the two inequalities can be used to characterize the separatedness of two clusters of random variables. Note that if indeed *Y *is from the same distribution as *F*_1_, namely, *F*_1 _= *F*_2_, then the equalities in (7) and (8) hold. In other words, a value of RAD close to zero indicates the cluster *J *is actually one cluster. Clearly RAD is bounded from above by 2. A value of RAD close to 2 indicates that the cluster *J *is comprised of two clusters *J*_1 _and *J*_2_. Summarizing our discussion above, we have the following result.

#### Selection criterion

A cluster with the largest value of RAD should be selected to split. If a cluster is less condensed, the RAD value will be larger. So the cluster with the largest RAD value will be the least condensed and thus should be selected for splitting.

### Evaluation measures

Suppose that *Z *= (*z*_*ij*_) is the *m *× *n *confusion matrix, where *z*_*ij *_is the number of data points which are predicted from cluster *C*_*i *_but in fact are from the true cluster *C*_*j*_. For generality, we use *m *and *n *where *m *and *n *can be different. But in our experiments, the number of actual clusters *k *is known, therefore *m *= *n *= *k*. mj=∑i=1mzij
 MathType@MTEF@5@5@+=feaafiart1ev1aaatCvAUfKttLearuWrP9MDH5MBPbIqV92AaeXatLxBI9gBaebbnrfifHhDYfgasaacH8akY=wiFfYdH8Gipec8Eeeu0xXdbba9frFj0=OqFfea0dXdd9vqai=hGuQ8kuc9pgc9s8qqaq=dirpe0xb9q8qiLsFr0=vr0=vr0dc8meaabaqaciaacaGaaeqabaqabeGadaaakeaacqWGTbqBdaWgaaWcbaGaemOAaOgabeaakiabg2da9maaqadabaGaemOEaO3aaSbaaSqaaiabdMgaPjabdQgaQbqabaaabaGaemyAaKMaeyypa0JaeGymaedabaGaemyBa0ganiabggHiLdaaaa@3BB4@ is the number of data points in the true cluster *j *and ni=∑j=1nzij
 MathType@MTEF@5@5@+=feaafiart1ev1aaatCvAUfKttLearuWrP9MDH5MBPbIqV92AaeXatLxBI9gBaebbnrfifHhDYfgasaacH8akY=wiFfYdH8Gipec8Eeeu0xXdbba9frFj0=OqFfea0dXdd9vqai=hGuQ8kuc9pgc9s8qqaq=dirpe0xb9q8qiLsFr0=vr0=vr0dc8meaabaqaciaacaGaaeqabaqabeGadaaakeaacqWGUbGBdaWgaaWcbaGaemyAaKgabeaakiabg2da9maaqadabaGaemOEaO3aaSbaaSqaaiabdMgaPjabdQgaQbqabaaabaGaemOAaOMaeyypa0JaeGymaedabaGaemOBa4ganiabggHiLdaaaa@3BB8@ is the number of data points in the predicted cluster *i*. Let *N *be the total number of data points.

One common measure of cluster quality is entropy. The entropy of predicted cluster *i *is defined as:

H(i)=−1log⁡k∑j=1kzijnilog⁡(zijni),
 MathType@MTEF@5@5@+=feaafiart1ev1aaatCvAUfKttLearuWrP9MDH5MBPbIqV92AaeXatLxBI9gBaebbnrfifHhDYfgasaacH8akY=wiFfYdH8Gipec8Eeeu0xXdbba9frFj0=OqFfea0dXdd9vqai=hGuQ8kuc9pgc9s8qqaq=dirpe0xb9q8qiLsFr0=vr0=vr0dc8meaabaqaciaacaGaaeqabaqabeGadaaakeaacqWGibascqGGOaakcqWGPbqAcqGGPaqkcqGH9aqpcqGHsisldaWcaaqaaiabigdaXaqaaiGbcYgaSjabc+gaVjabcEgaNjabdUgaRbaadaaeWbqaamaalaaabaGaemOEaO3aaSbaaSqaaiabdMgaPjabdQgaQbqabaaakeaacqWGUbGBdaWgaaWcbaGaemyAaKgabeaaaaGccyGGSbaBcqGGVbWBcqGGNbWzcqGGOaakdaWcaaqaaiabdQha6naaBaaaleaacqWGPbqAcqWGQbGAaeqaaaGcbaGaemOBa42aaSbaaSqaaiabdMgaPbqabaaaaOGaeiykaKIaeiilaWcaleaacqWGQbGAcqGH9aqpcqaIXaqmaeaacqWGRbWAa0GaeyyeIuoaaaa@55C8@

where *k *is the number of clusters.

The value of entropy ranges from 0 to 1. An entropy value of 0 means the cluster is comprised entirely of one class, while an entropy value near 1 implies that the cluster contains a uniform mixture of classes. The smaller the entropy value, the better the clustering performance.

Another measure of clustering we use is misclustering rate. Based on the confusion matrix, the accuracy *j*-th cluster is for *z*_*ij*_/*m*_*j*_. Since each true cluster contributes *m*_*j *_to the total N=∑i=1mmi+∑j=1nnj
 MathType@MTEF@5@5@+=feaafiart1ev1aaatCvAUfKttLearuWrP9MDH5MBPbIqV92AaeXatLxBI9gBaebbnrfifHhDYfgasaacH8akY=wiFfYdH8Gipec8Eeeu0xXdbba9frFj0=OqFfea0dXdd9vqai=hGuQ8kuc9pgc9s8qqaq=dirpe0xb9q8qiLsFr0=vr0=vr0dc8meaabaqaciaacaGaaeqabaqabeGadaaakeaacqWGobGtcqGH9aqpdaaeWaqaaiabd2gaTnaaBaaaleaacqWGPbqAaeqaaOGaey4kaScaleaacqWGPbqAcqGH9aqpcqaIXaqmaeaacqWGTbqBa0GaeyyeIuoakmaaqadabaGaemOBa42aaSbaaSqaaiabdQgaQbqabaaabaGaemOAaOMaeyypa0JaeGymaedabaGaemOBa4ganiabggHiLdaaaa@430A@ data points, its contribution has a weight *m*_*j*_/*N*. The global accuracy [20] is the weighted sum,

∑j=1nmjNzjjmj=∑j=1nzjjN.
 MathType@MTEF@5@5@+=feaafiart1ev1aaatCvAUfKttLearuWrP9MDH5MBPbIqV92AaeXatLxBI9gBaebbnrfifHhDYfgasaacH8akY=wiFfYdH8Gipec8Eeeu0xXdbba9frFj0=OqFfea0dXdd9vqai=hGuQ8kuc9pgc9s8qqaq=dirpe0xb9q8qiLsFr0=vr0=vr0dc8meaabaqaciaacaGaaeqabaqabeGadaaakeaadaaeWbqaamaalaaabaGaemyBa02aaSbaaSqaaiabdQgaQbqabaaakeaacqWGobGtaaWaaSaaaeaacqWG6bGEdaWgaaWcbaGaemOAaOMaemOAaOgabeaaaOqaaiabd2gaTnaaBaaaleaacqWGQbGAaeqaaaaakiabg2da9maaqahabaWaaSaaaeaacqWG6bGEdaWgaaWcbaGaemOAaOMaemOAaOgabeaaaOqaaiabd6eaobaaaSqaaiabdQgaQjabg2da9iabigdaXaqaaiabd6gaUbqdcqGHris5aaWcbaGaemOAaOMaeyypa0JaeGymaedabaGaemOBa4ganiabggHiLdGccqGGUaGlaaa@4DD4@

Then the misclustering rate is 1−∑j=1nzjjN
 MathType@MTEF@5@5@+=feaafiart1ev1aaatCvAUfKttLearuWrP9MDH5MBPbIqV92AaeXatLxBI9gBaebbnrfifHhDYfgasaacH8akY=wiFfYdH8Gipec8Eeeu0xXdbba9frFj0=OqFfea0dXdd9vqai=hGuQ8kuc9pgc9s8qqaq=dirpe0xb9q8qiLsFr0=vr0=vr0dc8meaabaqaciaacaGaaeqabaqabeGadaaakeaacqaIXaqmcqGHsisldaaeWaqaamaalaaabaGaemOEaO3aaSbaaSqaaiabdQgaQjabdQgaQbqabaaakeaacqWGobGtaaaaleaacqWGQbGAcqGH9aqpcqaIXaqmaeaacqWGUbGBa0GaeyyeIuoaaaa@3AE5@.

Since we do not know how to match up the predicted clusters with the true ones, *z*_*ij *_on the diagonal of the confusion matrix may not be the accurately predicted number of data. We use brute force to search for the best alignment between the predicted and the true clusters. The time complexity is *O*(*k*!) if there are *k *true clusters and *k *predicted clusters. This brute force approach is not a part of the algorithm itself, but is used to aid in a fair evaluation.

## Competing interests

The authors declare that they have no competing interests.

## Authors' contributions

XD and HP contributed to the theoretical development. YD and DW contributed to the experimentation and development of the computer code. All authors read and approved the final manuscript.

## Supplementary Material

Additional file 1 **The relationship between the number of runs and average entropy of the three algorithms on the Alon data**. Additional file 1 demonstrates that when the algorithms are run more times, the average entropy values of all the algorithms get more stable. In this figure, 500 genes were selected. Click here for file

Additional file 2 **The relationship between the number of runs and average entropy of the three algorithms on the SJCRH data**. Additional file 2 demonstrates that when the algorithms are run more times, the average entropy values of all the algorithms get more stable. In this figure, 1000 genes were selected. Click here for file

## References

[B1] Parsons L, Haque E, Liu H (2004). Subspace Clustering for High Dimensional Data: a Review. SIGKDD Explor Newsl.

[B2] Jörnsten R, Vardi Y, Zhang CH (2002). A Robust Clustering Method and Visualization Tool Based on Data Depth.

[B3] Garcia-Escudero LA, Gordaliza A (1999). Robustness Properties of k Means and Trimmed k Means. Journal of the American Statistical Association.

[B4] Tukey W (1975). Mathematics and the Picturing of Data. Proceedings of the International Congress of Mathematicians.

[B5] Oja H (1983). Descriptive Statistics for Multivariate Distributions. Statist Probab Lett.

[B6] Liu RY (1990). On a Notion of Data Depth Based upon Random Simplices. The Annals of Statistics.

[B7] Zuo Y, Serfling R (2000). General Notions of Statistical Depth Function. The Annals of Statistics.

[B8] Koshevoy G, Mosler K (1997). Zonoid Trimming for Multivariate Distributions. Annals of Statistics.

[B9] Zhang J (2002). Some Extensions of Tukey's Depth Function. Journal of Multivariate Analysis.

[B10] Chaudhuri P (1996). On a Geometric Notion of Quantiles for Multivariate Data. Journal of the American Statistical Association.

[B11] Vardi Y, Zhang CH (2000). The Multivariate L1-median and Associated Data Depth. Proc Natl Acad Sci USA.

[B12] Serfling R (2002). A Depth Function and a Scale Curve Based on Spatial Quantiles.

[B13] Koltchinskii VI (1997). M-estimation, Convexity and Quantiles. Ann Statistics.

[B14] Donoho DL, Huber P (1983). The Notion of Breakdown Point.

[B15] Hall P, Marron JS, Neeman A (2005). Geometric Representation of High Dimension, Low Sample Size Data. J R Statist Soc B.

[B16] Alon U, Barkai N, Notterman DA, Gish K, Ybarra S, Mack D, Levine AJ (1999). Broad Patterns of Gene Expression Revealed by Clustering Analysis of Tumor and Normal Colon Tissues Probed by Oligonucleotide Arrays. Proc Natl Acad Sci USA.

[B17] Yeoh EJ, Ross ME, Shurtleff SA, Williams WK, Patel D, Mahfouz R, Behm FG, Raimondi SC, Relling MV, Patel A, Cheng C, Campana D, Wilkins D, Zhou X, Li J, Liu H, Pui CH, Evans WE, Naeve C, Wong L, Downing JR (2002). Pediatric Lymphoblastic Leukemia by Gene Expression Profiling. Cancer Cell.

[B18] Ding Y, Wilkins D (2006). Improving the Performance of SVM-RFE to Select Genes in Microarray Data. BMC Bioinformatics.

[B19] Jörnsten R (2004). Clustering and Classification Based on the L1 Data Depth. Journal of Multivariate Analysis.

[B20] Ding C, He X (2002). Cluster Merging and Splitting in Hierarchical Clustering Algorithms. Proceedings of IEEE International Conference on Data Mining (ICDM'02).

